# Association of hypertriglyceridemic waist phenotype with renal function impairment: a cross-sectional study in a population of Chinese adults

**DOI:** 10.1186/s12986-020-00483-7

**Published:** 2020-08-05

**Authors:** Yun Qiu, Qi Zhao, Na Wang, Yuting Yu, Ruiping Wang, Yue Zhang, Shuheng Cui, Meiying Zhu, Xing Liu, Yonggen Jiang, Genming Zhao

**Affiliations:** 1 Department of Epidemiology, School of Public Health, Key Laboratory of Public Health Safety of Ministry of Education, Fudan University, Shanghai, 200032 China; 2 Songjiang District Center for Disease Control and Prevention, Shanghai, 201600 China

**Keywords:** Chronic kidney disease, Estimated glomerular filtration rate, Hypertriglyceridemic waist phenotype, Renal function decline

## Abstract

**Background:**

Hypertriglyceridemic waist (HTGW) phenotype has been suggested as a risk factor for chronic kidney disease (CKD). However, there is limited evidence on the relationship of triglyceride waist phenotypes with estimated glomerular filtration rate (eGFR) status and severity. Our aim was to explore the associations of triglyceride waist phenotypes with reduced eGFR and various decreased eGFR stages among Chinese adults.

**Methods:**

A population-based, cross-sectional study was conducted among Chinese participants aged 20–74 years from June 2016 to December 2017 in Shanghai, China. An eGFR value below 60 mL/min/1.73 m^2^ was defined as decreased eGFR. HTGW phenotype was defined as triglyceride (TG) ≥1.7 mmol/L and a waist circumference (WC) of ≥90 cm for men and ≥ 80 cm for women. We examined the association of triglyceride waist phenotypes with decreased eGFR risk using the weighted logistic regression models.

**Results:**

A total of 31,296 adults were included in this study. Compared with normal TG level/normal WC (NTNW) phenotype, normal TG level/enlarged WC (NTGW) and elevated TG level/enlarged WC (HTGW) phenotypes were associated with the increased risk of decreased eGFR. Multivariable-adjusted ORs (95% CI) associated with NTGW, elevated TG level/normal WC (HTNW), and HTGW phenotypes were 1.75 (1.41–2.18), 1.29 (0.99–1.68), and 1.99 (1.54–2.58), respectively. These associations between triglyceride waist phenotypes and decreased eGFR risk remained across almost all the subgroups, including sex, age, BMI, T2DM, and hypertension. HTGW phenotype was consistently positively associated with the risk of mildly and moderately decreased eGFR, but not with severely decreased eGFR risk.

**Conclusions:**

HTGW was consistently associated with the increased risk of decreased eGFR and various decreased eGFR stages, except for severely decreased eGFR. Further prospective studies are warranted to confirm our findings and to investigate the underlying biological mechanisms.

## Introduction

Chronic kidney disease (CKD) remains a main cause of morbidity and mortality worldwide, with increasing prevalence and incidence [1]. One of the characteristics of CKD is a decline of kidney function, which is assessed by estimated glomerular filtration rate (eGFR), a most commonly used marker [2]. The global prevalence of CKD and decreased eGFR (eGFR < 60 mL/min/1.73 m^2^) in general populations were 14.3 and 9.8%, respectively in 2016 [3]. The overall prevalence of CKD was 10.8% and decreased eGFR was 1.7% in the a general population of Chinese adults in 2012 [4]. Recent rapid increases in diabetes, hypertension, and obesity cases will contribute to the increase in CKD prevalence, eventually leading to higher burden of CKD and a bigger threat to public health in less developed regions [5]. Patients with kidney function decline were more likely to have increased risk of cardiovascular events [6], and death from cardiovascular diseases (CVD) [7].

Hypertriglyceridemic waist (HTGW) phenotype is defined by the simultaneous presence of elevated serum triglycerides (TG) level and increased waist circumference (WC). It was first proposed by Lemieux et al. [8], as an indicator of atherosclerosis and an effective tool to identify men who were at high risk of coronary artery disease (CAD). Because assessment of HTGW is relatively inexpensive and easy to acquire, a growing number of studies have shown that HTGW phenotype was associated with the increased risk of CVD [9], CAD [10], hypertension [11], prediabetes [12], and type 2 diabetes mellitus (T2DM) [13], as well as hyperuricemia [14].

Early identification of pertinent risk factors is needed for prevention and control of renal function decline and the development of CKD. In comparison to elevated TG or enlarged WC used alone, the HTGW phenotype is superior in evaluating excess visceral adiposity, and is also a useful clinical tool for identifying individuals with higher risk of abnormal metabolism [15]. Several studies have reported that HTGW phenotype is associated with an increased risk of CKD in adults aged ≥40 years old [16], in elderly (aged ≥60 years old) [17], and in relatively lean people (BMI < 24 kg/m^2^) [18]. However, existing evidence regarding the association of HTGW with CKD remains controversial. In a cross-sectional study, Ramezankhani et al. [19] found a positive association of HTGW with CKD only in women; while no significant association between HTGW and CKD was observed in the prospective study. A recent cross-sectional study has demonstrated that HTGW was related to CKD risk in women group but not in men group among adults aged 18 to 75 years old [20]. While another cross-sectional study in elderly participants reached an opposing conclusion [17]. Thus far, limited evidence suggest increased risk of decline renal function with HTGW [21]. Previous studies were limited by the lack of stratified analyses. Furthermore, no study has reported the relationship of HTGW with the various stages of decreased eGFR, which could represent the progression of CKD.

Using data from physical examinations and electronic medical records, we aimed to examine the association of HTGW and three other triglyceride waist phenotypes with risk of decreased eGFR in overall and subgroup population among Chinese adults. Additionally, we explored whether these phenotypes were associated with different stages of eGFR, including mildly, moderately, and severely decreased eGFR.

## Methods

### Study population

**Fig. 1:**
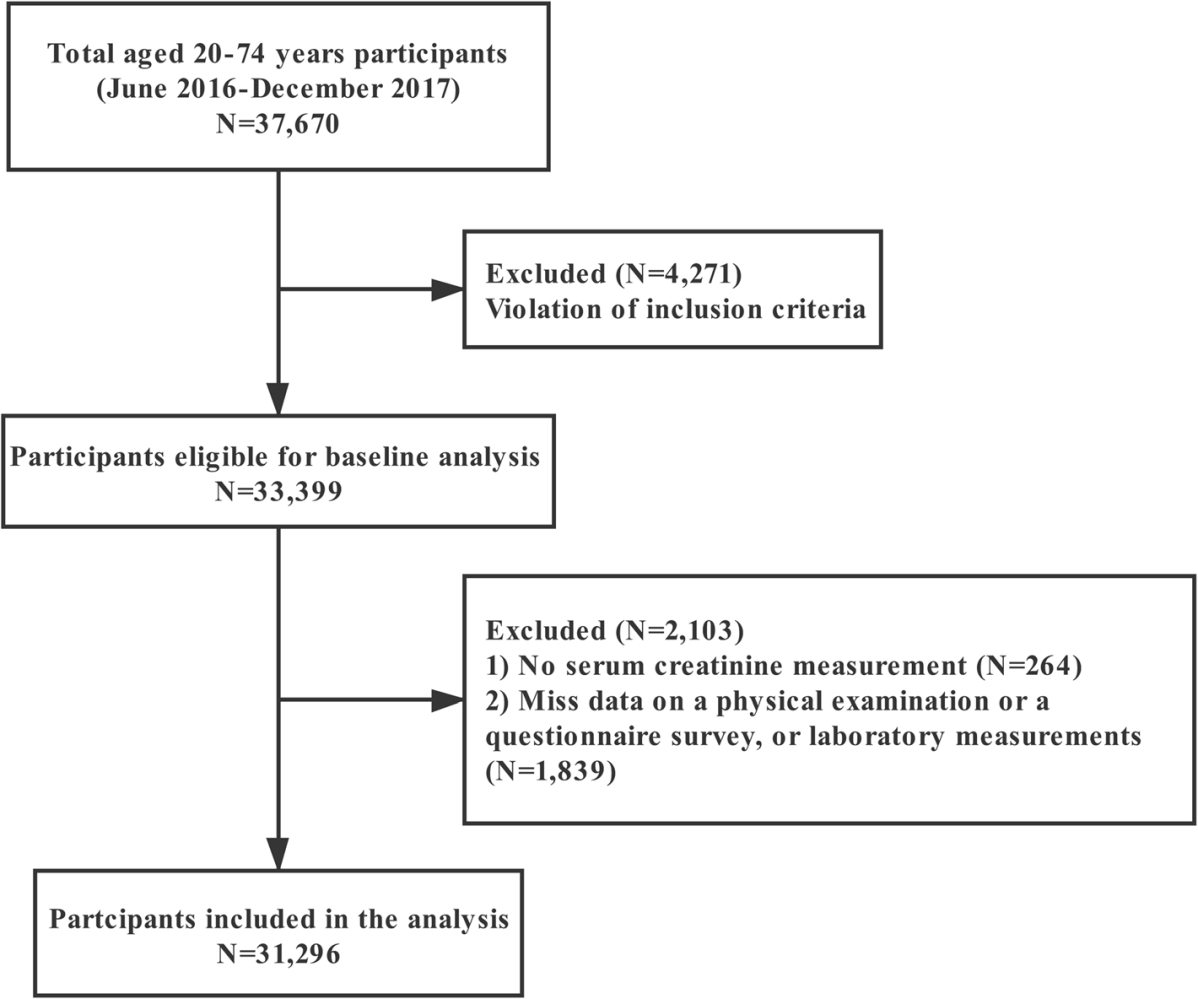
Flowchart of the study population

This population-based, cross-sectional study was conducted in Community Health Centers of Songjiang District, Shanghai, China from June 2016 to December 2017. Details of sample methods in this study have been described elsewhere [22]. Briefly, we used a multistage, stratified, clustered sampling method to collect health-related data from 31 neighborhood committees and 16 administrative villages in four study community sites, including Zhongshan, Xinqiao, Sheshan, and Maogang. Exclusion criteria were as follows: unable or unwilling to provide a written informed consent form; pregnancy; previously diagnosed critical illness, including cancer, stroke, CAD, cirrhosis, chronic hepatitis, cardiorespiratory failure, and hyper-or hypothyroidism; or have got organ transplantation or on dialysis therapy. For the present analysis, a total of 37,670 adults aged 20 to 74 years who were natives of Shanghai municipality or those have lived in Shanghai for at least 5 years were enrolled in the present study. Among these, we excluded participants who violated the inclusion criteria (*n* = 4271), who had no serum creatinine (Scr) measurement (*n* = 264) or had missing data on physical examination, questionnaire survey, or laboratory measurements (*n* = 1839). The final analysis included 31,296 participants (Fig. 1). The study protocol was approved by the Ethics Committee of Fudan University, School of Public Health (IRB#2016-04-0586) and complied with the principles of the Declaration of Helsinki. Informed written consent were obtained from all participants prior to data collection.

### Data collection

The general information of all study participants including sociodemographic characteristics (age, sex, marital status, educational level, and working status), self-reported history of chronic diseases (such as T2DM, hypertension, and cancer), and lifestyle factors (smoking status, alcohol consumption, and physical activity) was collected by trained interviewers through face-to-face interviews using a structured questionnaire. History of a diagnosis of T2DM, hypertension, cancer, stroke, CAD, cirrhosis, chronic hepatitis, cardiorespiratory failure, and hyper-or hypothyroidism, as well as receipt of organ transplantation or dialysis was obtained from electronic medical records. We used the International Physical Activity Questionnaire to assess physical activity. Smoking status was defined as > 1 cigarette per day and lasting > 6 months, and alcohol consumption was defined as alcohol intake at least 3 times per week and lasting for at least half a year. Smoking status and alcohol consumption have been classified as never, former, or current.

Anthropometric data were obtained from all participants, including height, weight, and waist circumference (WC), and were measured in duplicate when participants were wearing light clothing without shoes. The mean values of these measurements were calculated for further analysis. Body mass index (BMI) was defined as body weight in kilograms divided by height in meters squared (kg/m^2^). Blood pressure (BP) was consecutively measured three times using an electronic sphygmomanometer, and the mean values were used for analysis.

### Laboratory measurement

Prior to the investigation, all participants were asked to fast overnight for at least 8 h, and fasting venous blood specimens were collected to perform laboratory measurements in DiAn medical laboratory center. Serum total cholesterol, TG, high-density lipoprotein (HDL) cholesterol, and low-density lipoprotein (LDL) cholesterol levels were measured using an automatic biochemical analyzer (Roche Cobas C501). Fasting plasma glucose (FPG) level was measured by glycokinase method using Roche P800 biochemical analyzer. Scr level was measured using enzymatic methods by Roche C702 automatic biochemical analyzer. HbA1c level was determined using high pressure liquid chromatography (TOSOH G8 automatic biochemical analyzer).

### Kidney function assessment

The estimated glomerular fltration rate (eGFR) was calculated using the Chronic Kidney Disease Epidemiology Collaboration (CKD-EPI) equation for Chinese population [23]:

$$ \mathrm{eGFR}\left(\mathrm{mL}/\min /1.73{\mathrm{m}}^2\right)=141\times \min {\left(\mathrm{Scr}/\upkappa, 1\right)}^{\upalpha}\times \max {\left(\mathrm{Scr}/\upkappa, 1\right)}^{-1.209}\times {0.993}^{\mathrm{Age}}\times 1.018\left[\mathrm{if}\ \mathrm{female}\right] $$

where Scr is the serum creatinine (mg/dl), *k* is 0.7 for females and 0.9 for males, α is − 0.329 for females and − 0.411 for males, min indicates the minimum of Scr/*k* or 1, and max indicates the maximum of Scr/*k* or 1.

Decreased eGFR was defined as an eGFR value below 60 mL/min/1.73 m^2^. The CKD classification was in accordance with the National Kidney Foundation [2], and we classified GFR stages into 4 categories as follows: normal eGFR, ≥90 mL/min/1.73 m^2^; mildly decreased eGFR, 60–89 mL/min/1.73 m^2^; moderately decreased eGFR, 30–59 mL/min/1.73 m^2^; and severely decreased eGFR, 15–29 mL/min/1.73 m^2^. In addition, eGFR was evaluated using the Modification of Diet in Renal Disease (MDRD) Study Equation for Chinese population [24] in a sensitivity analysis:

$$ {\mathrm{eGFR}}_{\mathrm{MDRD}}\left(\mathrm{ml}/\min /1.73{\mathrm{m}}^2\right)=175\times \mathrm{serum}\ \mathrm{creatinine}{\left(\mathrm{mg}/\mathrm{dl}\right)}^{-1.154}\times {\mathrm{age}}^{-0.203}\times 0.742\left[\mathrm{if}\ \mathrm{female}\right] $$

### Definitions of triglyceride waist phenotype, T2DM, and hypertension

Participants were classified into four groups according to the following cut-off points [13]: (1) NTNW, normal serum TG level (< 1.7 mmol/L) and normal WC (< 90 cm for men and < 80 cm for women); (2) NTGW, normal serum TG level and enlarged WC (≥90 cm for men and ≥ 80 cm for women); (3) HTNW, elevated serum TG level (≥1.7 mmol/L) and normal WC; (4) HTGW, elevated serum TG level and enlarged WC. In the sensitivity analyses, HTGW phenotype was also defined as elevated serum TG level (≥2.0 mmol/L) along with enlarged WC (≥90 cm for men and ≥ 85 cm for women) [15], or a TG level ≥ 2.0 mmol/L and WC ≥90 cm for men or a TG level ≥ 1.5 mmol/L and WC ≥85 cm for women [25].

The definition of T2DM was in accordance with the American Diabetes Association criteria [26]: FPG level ≥ 7.0 mmol/L, HbA1c concentration ≥ 6.5%, or previously diagnosed type 2 diabetes mellitus (T2DM). The diagnosis of hypertension was according to the Seventh Joint National Committee Report on Detection, Evaluation, and Treatment of High Blood Pressure guidelines (JNC-7) [27]: systolic BP ≥140 mmHg, diastolic BP ≥90 mmHg, or previously diagnosed hypertension.

### Statistical analysis

We accounted for a complex sample survey design, and the results were weighted in the present study. Continuous variables were presented as means ± standard deviation (SD) or median with interquartile ranges. Categorical variables were expressed as number and percentage. We compared the differences between decreased eGFR and non-decreased eGFR using student’s t test or Mann-Whitney U test for comparisons of continuous data and χ^2^ test for categorical data. We used weighted logistic regression models to determine the association of triglyceride waist phenotypes with decreased eGFR, and odd ratios (ORs) and 95% confidence intervals (CIs) were calculated, with NTNW as the reference group. Multiple models were adjusted for age, sex (men vs. women), marital status (married vs. unmarried/divorced/widowed), educational level (0–6, 7–12, and > 12 years), working status (retired vs. not retired), smoking status (never, former, and current), alcohol consumption (never, former, and current), physical activity, BMI, hypertension, LDL cholesterol, HDL cholesterol, total cholesterol, and T2DM.

For sensitivity analyses, we repeated the analyses to examine the association of triglyceride waist phenotypes with decreased eGFR and estimated GFR by the use of the MDRD equation or redefined the triglyceride waist phenotypes on the basis of the previous recommended criteria. We performed stratified analyses and potential effect modifications by sex, age (< 60 years, ≥60 years), BMI (< 24 kg/m^2^, ≥24 kg/m^2^), and presence or absence of T2DM or hypertension. In addition, we investigated the associations of four phenotype groups with the severity of decreased eGFR, including mildly, moderately, and severely decreased eGFR with using weighted multinomial logistic regression models, respectively by treating the normal eGFR group as the control group, and the same confounding factors as above were adjusted for the analyses.

All analyses were performed using SAS 9.4 version (Institute Inc., Cary, NC, USA). *P* values of less than 0.05 (two-sided) were considered to be statistically significance.

## Results

### Baseline characteristics of subjects with or without decreased eGFR

Baseline characteristics of the study participants based on decreased eGFR status were shown in Table 1. Among 31,296 participants, the mean age of the study participants was 55.64 ± 11.35 years, 12,702 (40.59%) of them were men, 4247 (13.57%) had T2DM, 15,881 (50.74%) had hypertension. As expected, participants with decreased eGFR were more likely to have HTGW phenotype than those without decreased eGFR. Subjects with decreased eGFR had higher Scr, BMI, WC, BP, total cholesterol, TG, LDL cholesterol, and FPG levels, and a lower eGFR level in comparison with those in non-decreased eGFR subjects. In addition, less education, a higher proportion in past smokers and past drinkers were observed in decreased eGFR group than in non-decreased eGFR group. No significant differences in HDL cholesterol, marital status and physical activity were observed between the two groups.

**Table 1:** Characteristics of study participants by decreased eGFR

Variables	Total	Decreased eGFR	Non-decreased eGFR	***P*** value
Men/Women	12,702/18,594	350/479	12,352/18,115	0.333
Age, years	55.64 ± 11.35	64.10 ± 7.23	55.41 ± 11.36	< 0.001
Serum creatinine, mg/dl	0.80 ± 0.24	1.40 ± 0.96	0.78 ± 0.16	< 0.001
eGFR, ml/min/1.73 m^2^	92.75 ± 15.33	50.95 ± 10.13	93.88 ± 13.77	< 0.001
Body mass index, kg/m^2^	24.35 ± 3.34	25.40 ± 3.25	24.32 ± 3.34	< 0.001
Waist circumference, cm	81.50 ± 9.40	86.43 ± 9.24	81.36 ± 9.37	< 0.001
Systolic blood pressure, mmHg	133.29 ± 19.37	144.40 ± 20.91	133.00 ± 19.23	< 0.001
Diastolic blood pressure, mmHg	80.05 ± 10.52	81.95 ± 10.85	80.00 ± 10.51	< 0.001
Total cholesterol, mmol/L	4.94 ± 0.93	5.19 ± 1.00	4.94 ± 0.93	< 0.001
Triglycerides, mmol/L	1.35 (0.98–1.92)	1.53 (1.12–2.17)	1.34 (0.97–1.91)	< 0.001
HDL cholesterol, mmol/L	1.41 ± 0.36	1.39 ± 0.40	1.41 ± 0.35	0.106
LDL cholesterol, mmol/L	2.78 ± 0.83	2.97 ± 0.92	2.78 ± 0.83	< 0.001
Fasting glucose, mmol/L	4.72 (4.26–5.36)	4.82 (4.35–5.52)	4.72 (4.25–5.36)	< 0.001
Marital status, n (%)				0.597
Married	29,137 (93.10)	768 (92.64)	28,369 (93.11)	
Unmarried/Divorced/Widowed	2159 (6.90)	61 (7.36)	2098 (6.89)	
Educational level, years				< 0.001
≤ 6^a^	14,068 (44.95)	538 (64.90)	13,530 (44.41)	
7-12^a^	15,028 (48.02)	280 (33.78)	14,748 (48.41)	
≥ 13^a^	2200 (7.03)	11 (1.33)	2189 (7.18)	
Working status, n (%)				< 0.001
Not retired^a^	13,667 (43.67)	149 (17.97)	13,518 (44.37)	
Retired^a^	17,629 (56.33)	680 (82.03)	16,949 (55.63)	
Smoking status, n (%)				< 0.001
Never	23,917 (76.42)	645 (77.80)	23,272 (76.38)	
Former^a^	1048 (3.35)	51 (6.15)	997 (3.27)	
Current^a^	6331 (20.23)	133 (16.04)	6198 (20.34)	
Alcohol consumption, n (%)				0.002
Never	27,064 (86.48)	724 (87.33)	26,340 (86.45)	
Former^a^	282 (0.90)	16 (1.93)	266 (0.87)	
Current	3950 (12.62)	89 (10.74)	3861 (12.67)	
Physical activity, MET-min/week	2351.42 ± 591.27	2372.90 ± 593.80	2350.80 ± 591.20	0.290
Type 2 diabetes mellitus, n (%)	4247 (13.57)	167 (20.14)	4080 (13.39)	< 0.001
Hypertension, n (%)	15,881 (50.74)	634 (76.48)	15,247 (50.04)	< 0.001
Hypertriglyceridemic waist phenotype, n (%)				< 0.001
NTNW^a^	13,905 (44.43)	213 (25.69)	13,692 (44.94)	
NTGW^a^	7179 (23.00)	273 (32.93)	6924 (22.73)	
HTNW	4806 (15.36)	109 (13.15)	4697 (15.42)	
HTGW^a^	5388 (17.22)	234 (28.23)	5154 (16.92)	

Data are presented as mean ± SD, median (interquartile range), or number (%)

*eGFR* Estimated glomerular filtration rate, *HDL* High-density lipoprotein, *LDL* Low-density lipoprotein, *MET* Metabolic equivalent task, *NWNT* Normal triglyceride level (< 1.7 mmol/L) and normal waist circumference (< 90 cm for men and < 80 cm for women), *NTGW* Normal triglyceride level and enlarged waist circumference (≥90 cm for men and ≥ 80 cm for women), *HTNW* Elevated triglyceride level (≥1.7 mmol/L) and normal waist circumference, *HTGW* Elevated triglyceride level and enlarged waist circumference

^a^*p* <  0.05 between decreased eGFR and non-decreased eGFR

### Association of triglyceride waist phenotypes with decreased eGFR

Table 2 showed the ORs and 95% CIs for the association of decreased eGFR with triglyceride waist phenotypes. Compared with the NTNW phenotype, NTGW and HTGW phenotypes were associated with a higher risk of decreased eGFR after adjusting for age, sex, marital status, educational level, working status, smoking status, alcohol consumption, physical activity, BMI, hypertension, LDL cholesterol, HDL cholesterol, total cholesterol, and T2DM; whereas HTNW phenotype was not significantly associated with decreased eGFR risk. The multivariable-adjusted ORs for decreased eGFR risk associated with NTGW, HTNW, and HTGW phenotypes were 1.75 (95% CI, 1.41–2.18 *P* <  0.001), 1.29 (95% CI, 0.99–1.68, *P* = 0.060), and 1.99 (95% CI, 1.54–2.58, P <  0.001), respectively. We also performed three sensitivity analyses including the MDRD equation-based eGFR and redefining the triglyceride waist phenotypes to assess the robustness of our results, and found similar results in all sensitivity analyses (Tables 2 and 3).

**Table 2:** Odds ratios for decreased eGFR according to triglyceride level and waist circumference

Variables	Cases/Participants	Model 1^**a**^	*P* value	Model 2^**b**^	*P* value	Model 3^**c**^	*P* value
		OR (95% CI)		OR (95% CI)		OR (95% CI)	
Decreased eGFR							
NTNW	213/13,905	1.00		1.00		1.00	
NTGW	273/7197	1.94 (1.60–2.35)	< 0.001	1.94 (1.60–2.34)	< 0.001	1.75 (1.41–2.18)	< 0.001
HTNW	109/4806	1.55 (1.22–1.96)	< 0.001	1.55 (1.23–1.96)	< 0.001	1.29 (0.99–1.68)	0.060
HTGW	234/5388	2.54 (2.09–3.09)	< 0.001	2.55 (2.09–3.10)	< 0.001	1.99 (1.54–2.58)	< 0.001
*P*-trend		< 0.001		< 0.001		< 0.001	
Decreased eGFR_MDRD_							
NTNW	351/13,905	1.00		1.00		1.00	
NTGW	402/7197	1.81 (1.56–2.11)	< 0.001	1.82 (1.56–2.12)	< 0.001	1.79 (1.50–2.13)	< 0.001
HTNW	150/4806	1.24 (1.02–1.51)	0.029	1.24 (1.02–1.51)	0.029	1.11 (0.89–1.39)	0.348
HTGW	316/5388	2.07 (1.77–2.43)	< 0.001	2.09 (1.78–2.45)	< 0.001	1.88 (1.52–2.34)	< 0.001
*P*-trend		< 0.001		< 0.001		< 0.001	

*eGFR* Estimated glomerular filtration rate, *OR* Odds ratio, *CI* Confidence interval, *MDRD* Modification of Diet in Renal Disease, *NWNT* Normal triglyceride level (< 1.7 mmol/L) and normal waist circumference (< 90 cm for men and < 80 cm for women), *NTGW* Normal triglyceride level and enlarged waist circumference (≥90 cm for men and ≥ 80 cm for women), *HTNW* Elevated triglyceride level (≥1.7 mmol/L) and normal waist circumference, *HTGW* Elevated triglyceride level and enlarged waist circumference

^a^Adjusted for age and sex

^b^Adjusted for model 1 covariates plus marital status, educational level, working status, smoking status, alcohol consumption, and physical activity

^c^Adjusted for model 2 covariates plus body mass index, hypertension, low-density lipoprotein (LDL) cholesterol, high-density lipoprotein (HDL) cholesterol, total cholesterol, and T2DM

**Table 3:** Odds ratios for decreased eGFR at different levels of triglyceride level and waist circumference

Variables	Cases/Participants	Model 1^**a**^	*P* value	Model 2^**b**^	*P* value	Model 3^**c**^	*P* value
		OR (95% CI)		OR (95% CI)		OR (95% CI)	
Definition 1							
NTNW1	317/18,318	1.00		1.00		1.00	
NTGW1	250/5808	1.80 (1.52–2.14)	< 0.001	1.80 (1.51–2.14)	< 0.001	1.69 (1.38–2.07)	< 0.001
HTNW1	107/4228	1.52 (1.22–1.90)	< 0.001	1.52 (1.21–1.90)	< 0.001	1.35 (1.04–1.76)	0.026
HTGW1	155/2942	2.78 (2.28–3.40)	< 0.001	2.80 (2.29–3.41)	< 0.001	2.42 (1.84–3.17)	< 0.001
*P*-trend		< 0.001		< 0.001		< 0.001	
Definition 2							
NTNW2	269/16,019	1.00		1.00		1.00	
NTGW2	196/4548	1.89 (1.56–2.28)	< 0.001	1.88 (1.55–2.27)	< 0.001	1.76 (1.41–2.19)	< 0.001
HTNW2	155/6527	1.50 (1.22–1.84)	< 0.001	1.49 (2.21–1.83)	< 0.001	1.26 (1.00–1.60)	0.054
HTGW2	209/4202	2.57 (2.12–3.11)	< 0.001	2.58 (2.13–3.12)	< 0.001	2.10 (1.62–2.71)	< 0.001
*P*-trend		< 0.001		< 0.001		< 0.001	

*eGFR* Estimated glomerular filtration rate, *OR* Odds ratio, *CI* Confidence interval, *NWNT1* Normal triglyceride level (< 2.0 mmol/L) and normal waist circumference (< 90 cm for men and < 85 cm for women), *NTGW1* Normal triglyceride level and enlarged waist circumference (≥90 cm for men and ≥ 85 cm for women), *HTNW1* Elevated triglyceride level (≥2.0 mmol/L) and normal waist circumference, *HTGW1* Elevated triglyceride level and enlarged waist circumference, *NWNT2* Normal triglyceride level (< 2.0 mmol/L for men and < 1.5 mmol/L for women) and normal waist circumference (< 90 cm for men and < 85 cm for women), *NTGW2* Normal triglyceride level and enlarged waist circumference (≥90 cm for men and ≥ 85 cm for women), *HTNW2* Elevated triglyceride level (≥2.0 mmol/L for men and ≥ 1.5 mmol/L for women) and normal waist circumference, *HTGW2* Elevated triglyceride level and enlarged waist circumference

^a^Adjusted for age and sex

^b^Adjusted for model 1 covariates plus marital status, educational level, working status, smoking status, alcohol consumption, and physical activity

^c^Adjusted for model 2 covariates plus body mass index, hypertension, low-density lipoprotein (LDL) cholesterol, high-density lipoprotein (HDL) cholesterol, total cholesterol, and T2DM

We further examined the association of triglyceride waist phenotypes with decreased eGFR in different subgroups of sex, age, BMI, T2DM, and hypertension in Table 4. The associations between NTGW, HTNW, and HTGW phenotypes and the risk of decreased eGFR remained consistent across almost all subgroups. The strongest positive association of HTGW phenotype with decreased eGFR was found in the subgroup of presence of T2DM (OR 2.60, 95% CI 1.38–4.89). No significant interaction effect was observed between the triglyceride waist phenotypes and all subgroup variables in decreased eGFR risk.

**Table 4:** Odds ratios for decreased eGFR according to triglyceride level and waist circumference by various subpopulations

Subpopulation	Cases/Participants	NTNW	NTGW	HTNW	HTGW	P-trend	P-interaction
Sex groups							0.637
Men	350/12,702	1.00	1.66 (1.19–2.33)	1.30 (0.92–1.85)	1.76 (1.17–2.64)	0.016	
Women	479/18,594	1.00	1.83 (1.36–2.45)	1.21 (0.80–1.85)	2.27 (1.61–3.20)	< 0.001	
Age, years							0.642
< 60	197/18,111	1.00	2.00 (1.28–3.12)	1.33 (0.82–2.17)	1.96 (1.17–3.26)	0.052	
≥ 60	632/13,185	1.00	1.74 (1.35–2.23)	1.29 (0.94–1.78)	2.09 (1.54–2.83)	< 0.001	
BMI, kg/m^2^							0.431
< 24	288/14,965	1.00	2.09 (1.48–2.95)	1.29 (0.86–1.93)	1.84 (1.10–3.08)	0.008	
≥ 24	541/16,331	1.00	1.73 (1.28–2.34)	1.37 (0.93–2.02)	2.06 (1.47–2.89)	< 0.001	
Presence of T2DM							0.412
No	662/27,049	1.00	1.63 (1.28–2.07)	1.29 (0.96–1.73)	2.00 (1.50–2.67)	< 0.001	
Yes	167/4247	1.00	2.90 (1.65–5.12)	1.49 (0.76–2.89)	2.60 (1.38–4.89)	0.098	
Presence of hypertension							0.542
No	195/15,415	1.00	1.58 (1.04–2.39)	0.92 (0.52–1.60)	1.70 (0.99–2.92)	0.155	
Yes	634/15,881	1.00	1.84 (1.42–2.38)	1.46 (1.07–1.98)	2.14 (1.58–2.88)	< 0.001	

All models adjusted for age, sex, marital status, educational level, working status, smoking status, alcohol consumption, physical activity, body mass index, hypertension, low-density lipoprotein (LDL) cholesterol, high-density lipoprotein (HDL) cholesterol, total cholesterol, and T2DM

*eGFR* estimated glomerular filtration rate, *BMI* Body mass index, *T2DM* Type 2 diabetes mellitus, *NWNT* Normal triglyceride level (< 1.7 mmol/L) and normal waist circumference (< 90 cm for men and < 80 cm for women), *NTGW* Normal triglyceride level and enlarged waist circumference (≥90 cm for men and ≥ 80 cm for women), *HTNW* Elevated triglyceride level (≥1.7 mmol/L) and normal waist circumference, *HTGW* Elevated triglyceride level and enlarged waist circumference

### Association of different triglyceride waist phenotypes with mildly, moderately, and severely decreased eGFR

The multivariable-adjusted ORs for mildly, moderately, and severely decreased eGFR according to triglyceride waist phenotypes were present in Table 5. The number of participants with normal eGFR, mildly decreased eGFR, moderately decreased eGFR, and severely decreased eGFR were 19,538, 10,929, 785, and 30, respectively. After adjusting for potential confounders, HTGW phenotype was positively associated with mildly decreased eGFR risk, whereas no significant association of NTGW and HTNW phenotypes with mildly decreased eGFR was found. Risk of moderately decreased eGFR was higher for subjects with NTGW and HTGW phenotypes, but not for subjects with HTNW phenotype, as compared to subjects with NTNW phenotype. This was consistent with the results of primary analyses among all subjects. No significant association was found between NTGW, HTNW, and HTGW phenotypes and the risk of severely decreased eGFR.

**Table 5:** Odds ratios for mildly, moderately, and severely decreased eGFR according to triglyceride level and waist circumference

	NTNW	NTGW	***P*** value	HTNW	***P*** value	HTGW	***P*** value	P-trend
**Mildly decreased eGFR**								
Number of participants	4368	2784		1684		2093		
Model 1^a^	1.00	1.12 (1.05–1.20)	< 0.001	1.16 (1.08–1.25)	< 0.001	1.26 (1.18–1.36)	< 0.001	< 0.001
Model 2^b^	1.00	1.13 (1.06–1.21)	< 0.001	1.16 (1.08–1.26)	< 0.001	1.28 (1.19–1.37)	< 0.001	< 0.001
Model 3^c^	1.00	1.05 (0.97–1.13)	0.262	1.07 (0.98–1.17)	0.145	1.13 (1.02–1.24)	0.020	0.020
**Moderately decreased eGFR**								
Number of participants	200	260		99		226		
Model 1^a^	1.00	2.07 (1.70–2.53)	< 0.001	1.60 (1.25–2.05)	< 0.001	2.90 (2.37–3.56)	< 0.001	< 0.001
Model 2^b^	1.00	2.08 (1.70–2.54)	< 0.001	1.60 (1.25–2.05)	< 0.001	2.93 (2.39–3.60)	< 0.001	< 0.001
Model 3^c^	1.00	1.81 (1.44–2.27)	< 0.001	1.32 (1.00–1.75)	0.051	2.23 (1.70–2.92)	< 0.001	< 0.001
**Severely decreased eGFR**								
Number of participants	7	10		6		7		
Model 1^a^	1.00	2.01 (0.74–5.48)	0.173	2.79 (0.93–8.34)	0.066	2.31 (0.79–6.78)	0.129	0.084
Model 2^b^	1.00	2.06 (0.75–5.64)	0.159	2.86 (0.95–8.55)	0.061	2.35 (0.80–6.90)	0.121	0.078
Model 3^c^	1.00	1.50 (0.48–4.71)	0.487	1.57 (0.45–5.49)	0.481	1.10 (0.28–4.33)	0.890	0.938

*eGFR* Estimated glomerular filtration rate, *NWNT* Normal triglyceride level (< 1.7 mmol/L) and normal waist circumference (< 90 cm for men and < 80 cm for women), *NTGW* Normal triglyceride level and enlarged waist circumference (≥90 cm for men and ≥ 80 cm for women), *HTNW* Elevated triglyceride level (≥1.7 mmol/L) and normal waist circumference, *HTGW* Elevated triglyceride level and enlarged waist circumference

^a^Adjusted for age and sex

^b^Adjusted for model 1 covariates plus marital status, educational level, working status, smoking status, alcohol consumption, and physical activity

^c^Adjusted for model 2 covariates plus body mass index, hypertension, low-density lipoprotein (LDL) cholesterol, high-density lipoprotein (HDL) cholesterol, total cholesterol, and T2DM

## Discussion

In this large population-based, cross-sectional study, we explored the association of triglyceride waist phenotypes with decreased eGFR (< 60 mL/min/1.73 m^2^) in the overall population and across a variety of subgroups. We found that HTGW phenotype was associated an increased risk of decreased eGFR; in addition, HTGW phenotype was significantly associated with the progression of renal function decline except for severely decreased eGFR. To the best of our knowledge, this is the first study to examine the association of HTGW phenotype with different stages of renal function in Chinese adults.

HTGW phenotype was found to have a similar predictive power with metabolic syndrome (MetS), and may be more easily applicable than MetS [28]. A recent cross-sectional study has shown that the HTGW phenotype, and not the common indices (including WC, waist-to-hip ratio, and waist-to-height ratio), was associated with a higher risk of CKD [18]. The relationship between triglyceride waist phenotypes and CKD has been extensively reported in many cross-sectional studies. However, most studies were conducted in with a limited sample size [16–20] and findings were inconsistent. In a middle-aged or older population, compared with NTNW phenotype, NTGW/HTNW and HTGW phenotypes were associated with higher risk of CKD [16]. Results regarding association of triglyceride waist phenotypes with CKD in the cross-sectional study and prospective study were not identical [19]. The reasons for the conflicting results may likely be due to the difference of population characteristics, the number of participants, inclusion of confounding factors, and methods of eGFR calculation. Ramezankhani et al. [19] and our study estimated GFR using the CKD-EPI equation, which was a more accurate method of renal function than the MDRD Equation in routine clinical practice. While many previous studies [16–18, 20] used the MDRD Equation for estimating GFR, a most common method for estimating GFR.

The decline in eGFR was related with the increased risk of mortality and end-stage renal disease (ESRD) [29]. Only a few studies have determined the association of decreased eGFR risk with triglyceride waist phenotypes [21], and shown that HTGW was associated with abnormal renal function among both Chinese and Australian subjects. In the present large Chinese population, we also observed similar findings in overall and stratified populations; NTGW phenotype was also associated with an increased risk of reduced eGFR. In the stratified analyses, the independent positive association of NTGW and HTGW phenotypes with reduced eGFR risk still persisted across almost all subgroups; a strongest association between HTGW phenotype and decreased eGFR risk was found in the subgroup of presence of T2DM, suggesting the predictive power of HTGW for decreased eGFR might be better for T2DM patients. Our findings suggest that each of the triglyceride waist phenotypes may play different roles on renal function decline. This lack of evidence may be explained by differences of NTGW and HTNW phenotype on decreased eGFR, and it is in line with a previous study reporting associations with CKD in men [17]. TG was considered to be a useful marker of visceral obesity with a given WC [30]. Additionally, compared with NTGW or HTNW phenotype, HTGW phenotype was a more stable and stronger risk factor for renal function decline. Thus, our data provide evidence that HTGW phenotype might play an important role in the development of renal function impairment.

Corresponding studies on the role of triglyceride waist phenotypes on the development of kidney dysfunction remain scarce. Most studies were performed to examine the association of HTGW phenotype with CKD risk, but were not involved in CKD progression. In the present study, we found that NTGW and HTGW phenotypes were independently associated with mildly decreased eGFR, or moderately decreased eGFR risk, except for the NTGW-mildly decreased eGFR association. No significant association between any of the phenotypes and severely decreased eGFR was found. Underlying mechanisms of these associations are still unknown, the null association of triglyceride waist phenotypes with severely decreased eGFR may be due to the small number of subjects. Thus, our findings suggest that HTGW phenotype may be an independent risk factor for the development of renal function impairment, prevention and that control of HTGW may be an effective measure to attenuate the risk of the progression of renal function decline. HTGW was closely related to visceral obesity, which may result in fat accumulation in kidney [30, 31]. Excess accumulation of fat on the kidney, which is related to oxidative stress and inflammation response, may impair the kidney and contribute to an unfavorable renal hemodynamic profile [32]. CKD patients have a lower activity of lipoprotein lipase and hepatic TG lipase, which likely leads to the development of hypertriglyceridemia, and subsequently to augment renal impairment [33, 34]. Further elucidation of the mechanism for the role of HTGW on renal function or CKD is still warranted.

The strengths of the present study include a large-scale population-based study, enrollment of participants living in both urban and rural areas, and the extensive adjustment for potential confounders, including demographic, lifestyle, anthropometric, and clinical factors. Several potential limitations of our study also should be considered. Firstly, the causal relationship could not be determined due to the nature of cross-sectional study design in our analyses. Secondly, all study participants were from only one district of Shanghai, China, and the generalizability of our results may be limited. Further studies should be conducted in more diverse regions. Thirdly, although many covariates were adjusted for in our analyses, the present study still lacks data on medication treatment, dietary, and genetic factors, and so on. Lastly, despite the overall relatively large sample size, only a few participants were in the subpopulations, especially for severely decreased eGFR, which resulted in wide CIs for the effect estimates and inaccurate results.

## Conclusions

In conclusion, HTGW phenotype was significantly associated with an increased risk of decreased eGFR in the overall study population, and remained consistent across all subgroups, which suggests that the HTGW phenotype may be a stable risk factor for the decline of kidney function. We also found that the HTGW phenotype was significantly associated with higher risks of mildly and moderately decreased eGFR, but not with severely decreased eGFR. These findings underscore the importance of preventing and controlling the HTGW phenotype, which may reduce the risk of kidney function decline or even the progression of CKD.

## Data Availability

The dataset used and analyzed during the current study is available from the corresponding author on reasonable request.

## Abbreviations

BMI: Body mass index
BP: Blood pressure
CAD: Coronary artery disease
CIs: Confidence intervals
CKD: Chronic kidney disease
CKD-EPI: Chronic Kidney Disease Epidemiology Collaboration
CVD: Cardiovascular diseases
eGFR: Estimated glomerular filtration rate
ESRD: End-stage renal disease
FPG: Fasting plasma glucose
HDL: High-density lipoprotein
HTGW: Hypertriglyceridemic waist
HTNW: Elevated TG level/normal WC
LDL: Low-density lipoprotein
MDRD: Modification of Diet in Renal Disease
MetS: Metabolic syndrome
NTGW: Normal TG level/enlarged WC
NTNW: Normal TG level/normal WC
ORs: Odd ratios
Scr: Serum creatinine
TG: Triglycerides
T2DM: Type 2 diabetes mellitus
WC: Waist circumference
